# Electrocardiographic changes during sustained normobaric hypoxia in patients after myocardial infarction

**DOI:** 10.1038/s41598-023-43707-5

**Published:** 2023-10-14

**Authors:** Tilmann Kramer, Jan-Niklas Hoenemann, Henning Weis, Fabian Hoffmann, Stephan Rosenkranz, Stephan Baldus, Martin Hellmich, Benjamin D. Levine, Jens Jordan, Jens Tank, Ulrich Limper

**Affiliations:** 1 Cardiovascular Aerospace Medicine, German Aerospace Center, Cologne, Germany; 2 Department of Internal Medicine III, University Hospital of Cologne, Cologne, Germany; 3 Department of Nuclear Medicine, University Hospital of Cologne, Cologne, Germany; 4 Institute of Medical Statistics and Computational Biology, Faculty of Medicine and University Hospital of Cologne, University of Cologne, Cologne, Germany; 5 Division of Cardiology, UT Southwestern Medical Center, 5323 Harry Hines Blvd, Dallas, TX 75390 USA; 6 Director, Institute for Exercise and Environmental Medicine, Texas Health Presbyterian Hospital, Dallas, 7232 Greenville Ave, Dallas, TX 75231 USA; 7 Institute of Aerospace Medicine, German Aerospace Center, Linder Hoehe, 51147 Cologne, Germany; 8 Chair of Aerospace Medicine, University of Cologne, Cologne, Germany; 9 Department of Anaesthesiology and Intensive Care Medicine, Cologne-Merheim Medical Center, University of Witten Herdecke, Cologne, Germany

**Keywords:** Cardiology, Diseases, Medical research, Risk factors, Signs and symptoms

## Abstract

The safety of prolonged high-altitude stays and exercise for physically fit post-myocardial infarction (MI) patients is unclear. Myocardial tissue hypoxia and pulmonary hypertension can affect cardiac function and electrophysiology, possibly contributing to arrhythmias. We included four non-professional male athletes, clinically stable after left ventricular MI (three with ST-segment elevation MI and one with non-ST-segment elevation MI) treated with drug-eluting stents for single-vessel coronary artery disease. Oxygen levels were reduced to a minimum of 11.8%, then restored to 20.9%. We conducted electrocardiography (ECG), ergometry, and echocardiography assessments in normoxic and hypoxic conditions. With an average age of 57.8 ± 3.3 years and MI history 37 to 104 months prior, participants experienced a significant increase in QTc intervals during hypoxia using Bazett’s (from 402 ± 13 to 417 ± 25 ms), Fridericia’s (from 409 ± 12 to 419 ± 19 ms), and Holzmann's formulas (from 103 ± 4 to 107 ± 6%) compared to normoxia. This effect partially reversed during recovery. Echocardiographic signs of pulmonary hypertension during normobaric hypoxia correlated significantly with altered QTc intervals (p < 0.001). Despite good health and complete revascularization following MI, susceptibility to hypoxia-induced QTc prolongation and ventricular ectopic beats persists, especially during physical activity. MI survivors planning high-altitude activities should consult cardiovascular specialists with high-altitude medicine expertise.

## Introduction

Altitude exposure exceeding 4200 m warrants careful consideration in patients with ischemic heart disease^1^, however data is limited. Adverse consequences, like myocardial tissue hypoxia and pulmonary hypertension (PH), could impact cardiac function, electrophysiology, and predispose to arrhythmias. Sympathetic activation and cellular transmembrane potassium shifts exacerbate arrhythmia risk, especially at very high altitudes. Patients post myocardial infarction may be prone to ventricular arrhythmias depending on revascularization^2^, concomitant pharmacotherapy, left ventricular impairment, and myocardial scarring^3^.

**Figure 1:**
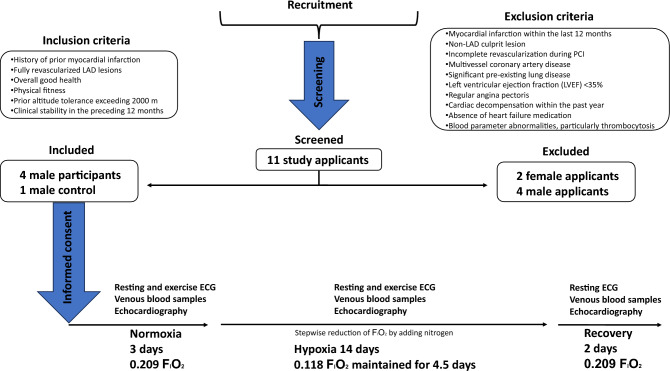
Visual representation of the study protocol.

We evaluated the safety and impact of normobaric hypoxia on both resting and exercise electrocardiograms (ECG) in patients with previous myocardial infarction. Normobaric hypoxia, simulating high-altitude conditions and replicating high-altitude pulmonary hypertension (PH)^1,4^, was equivalent to an altitude of approximately 4500 m. Furthermore, we performed echocardiography and assessed whether the alterations in ECG are associated with hypoxia-induced PH (Fig. 1).

Inclusion criteria encompassed patients with a history of prior myocardial infarction, fully revascularized left anterior descending (LAD) lesions, overall good health, physical fitness, and prior altitude tolerance exceeding 2000 m. Patients were clinically stable in the preceding 12 months and had previously tolerated altitudes of > 2000 m.

Exclusion criteria covered myocardial infarction within the last 12 months, non-LAD culprit lesion, incomplete revascularization during percutaneous coronary intervention (PCI), multivessel coronary artery disease, significant pre-existing lung disease, left ventricular ejection fraction (LVEF) < 35%, regular angina pectoris, cardiac decompensation within the past year, absence of heart failure medication, and blood parameter abnormalities, particularly thrombocytosis.

In the DLR:envihab hypoxia module (https://www.dlr.de/envihab/en/desktopdefault.aspx/), we gradually decreased atmospheric oxygen (FIO2) to a minimum of 11.8% (hypoxia), which equals the oxygen content at ≈ 4500 m, over a period of 14 days. FIO2 of 11.8% was maintained for 4.5 days followed by return to normoxia within two days. Subjects received a standard normocaloric diet with four meals per day. Following a structured daily schedule, subjects woke up at 7 a.m., spent 15 h awake performing light activities, and went to bed at 10 p.m. Each subject completed 60 min of aerobic exercise daily on a treadmill or bicycle. We obtained 12-lead resting ECG daily. Transthoracic echocardiography was performed on the second day of acclimatization and daily at 11.8% FIO_2_, and during the first two days of recovery at 20.9% FIO_2_ to assess echocardiographic signs of PH (*TR dPmax* tricuspid regurgitation jet maximal pressure gradient, *PVAT* pulmonary velocity acceleration time, *RA* right atrium area, *TR Vmax* maximal tricuspid regurgitation velocity). We submitted participants to exercise ECG with 25 W increments until exhaustion on a bicycle ergometer under normoxia, and under 17.5% FIO_2_ (~ 1500 m) but for safety reasons not during more intense hypoxia.

The study, including all experimental protocols, was registered (DRKS00013772), approved by the local ethics committee of the North Rhine Medical Association in Düsseldorf, Germany (“Ärztekammer Nordrhein”) and conducted in accordance with the Declaration of Helsinki after obtaining informed consent from all subjects. All methods were carried out in accordance with relevant guidelines and regulations.

Results are presented as mean ± standard deviation. Pearson correlation between any two variables were calculated and accompanied by a "Wald-type" p-value based on bootstrap standard errors. Differences in mean values over conditions were evaluated by linear mixed models with fixed effect condition and random effect subject. Differences between paired measurements were tested by the (exact) Wilcoxon signed-rank test. Additionally, Friedman test was applied to assess significant differences among multiple related groups with non-normally distributed dependent variables.

Of the initially screened 11 patients, we included four male recreational athletes (age, 54–63 years; body mass index, 21–25 kg/m^2^; VO_2_ max, 32–43 mL/kg per minute) who were in clinically stable conditions 37 to 104 months following left ventricular myocardial infarction. Three participants had experienced ST-segment-elevation myocardial infarction (STEMI), while one had a history of non-ST-segment-elevation myocardial infarction (NSTEMI). In each participant, effective revascularization was achieved by subsequent drug-eluting stent intervention, targeting an isolated stenosis within the mid-to-ostial segment of the LAD artery. Two women were excluded, one due to thrombocytosis and the other due to right ventricular involvement^5^. An additional male participant (aged 60 years), who was athletically active and had no previous history of myocardial infarction, was included as a control. Transthoracic echocardiography revealed distinct left ventricular wall motion abnormalities and corresponding LVEF for each patient: Patient 1 presented with apical, anteroseptal, and anterolateral akinesis (LVEF = 50%); Patient 2 showed apical, septal, anterior, and apical inferior akinesis (LVEF = 36%); Patient 3 exhibited apical and apical septal hypokinesis (LVEF > 50%); and Patient 4 had no abnormalities (EF = 58%). Following myocardial infarction, all participants received standard medical treatment, including aspirin for two out of four, marcumar for one out of four, anti-Xa-antagonist for one out of four, statins for all four, ACE inhibitors/AT1 antagonist for all four, azetidinone for one out of four, β-blockers for one out of four, and dihydropiyridine for one out of four^5^.

**Figure 2:**
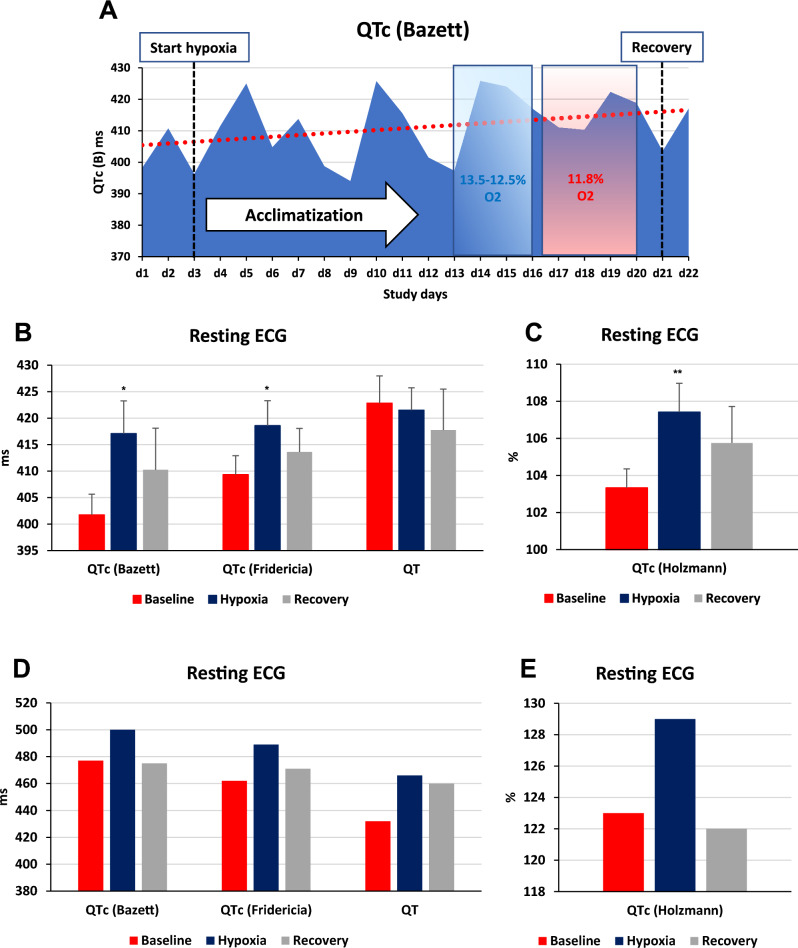

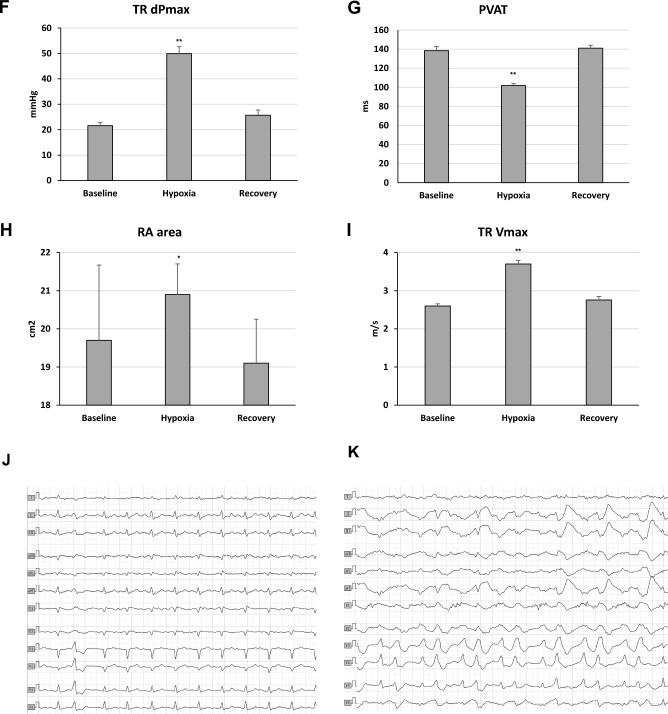
Resting electrocardiographic measures. (**A**) QTc (Bazett) interval during the hypoxia study. A 3-day normoxia phase was followed by an acclimatization phase with increasing hypoxia levels to a minimum of 11.8% FIO_2_ between the study days 16 and 20. Oxygen content was gradually increased back to normal during a 2-day recovery phase. Changes in standard 12-lead resting electrocardiogram intervals at baseline (normoxia), hypoxia and recovery (normoxia) of four individuals after myocardial infarction. (**B**) QTc(B) = QT correction using Bazett's formula, QTc(B) = QT/√RR; QTc(F) = QT correction using Fridericia's formula, QTc(F) = QT/3√RR; QT interval. (**C**) QTr(H) = relative QT interval by using Holzmann’s correction formula, QTr(H) = QT/(0.39 × √RR) × 100%. Changes in standard 12-lead resting electrocardiogram intervals at baseline (normoxia), hypoxia and recovery (normoxia) of one healthy control individual. (**D**) QTc(B) = QT correction using Bazett's formula, QTc(B); QTc(F), QTc(F) = QT/3√RR; QT interval. (**E**) QTr(H). Echocardiographic variables of right heart strain at baseline (normoxia), hypoxia and recovery (normoxia). (**F**) TR dPmax, tricuspid regurgitation jet maximal pressure gradient (mmHg); (**G**) PVAT, pulmonary velocity acceleration time (ms); (**H**) RA, right atrium area (cm^2^); (**I**) TR Vmax, maximal tricuspid regurgitation velocity (m/s). Exercise electrocardiographic measures. (**J**) ECG during exercise of one individual at 250 Watts under normoxia with a single ventricular extrasystolic beat; 150 bpm; 50 mm/s. (**K**) ECG during exercise of the same subject at 250 Watts in 17.5% oxygen illustrating multiple ventricular extrasystolic beats including bigemini and couplets; 124 bpm; 50 mm/s. No symptoms were reported. Columns represent means ± standard error of mean and corresponding p-values between different conditions (*p < 0.05; **p < 0.01).

In hypoxia, 12-lead ECG revealed statistically significant QTc interval prolongations using Bazett’s (from 402 ± 13 to 417 ± 25 ms), Fridericia's (from 409 ± 12 to 419 ± 19 ms), and Holzmann’s (from 103 ± 4 to 107 ± 6%) formula compared to normoxia (Fig. 2A–C). This effect was partially reversed during recovery. The healthy control demonstrated similar changes in QTc intervals during both hypoxia and recovery (Fig. 2D–E).

Serum potassium (baseline 4.56 ± 0.19; hypoxia 4.73 ± 0.23 mmol/L; p = 0.097) and magnesium levels (baseline 0.80 ± 0.03; hypoxia 0.89 ± 0.06 mmol/L; p = 0.028) increased within normal ranges during hypoxia. Normobaric hypoxia-induced PH, which subsequently reversed during the return to normoxia (Fig. 2F–I), without causing any changes in right atrial pressure.

Multiple echocardiographic indicators of PH demonstrated significant correlations with the altered QTc intervals [p < 0.001 for PVAT, TR Vmax, and TR dPmax with QTc (B), QTr (H), and QTc (F); p < 0.05 for RA with QTr (H) and QTc (F)]. LVEF improved significantly during and after hypoxia exposure (p = 0.0046, Friedman test, n = 4), with baseline, hypoxia, and recovery mean values of 51.2 ± 12%, 58.8 ± 16%, and 62.6 ± 13%, respectively^5^. Additionally, diastolic parameters such as the E/e′ ratio, showed a decreasing trend (p = 0.2731, Friedman test, n = 4), with baseline mean values at 8.0 ± 1.8, declining to 7.0 ± 1.3 during hypoxia, and further to 6.4 ± 1.4 during recovery. No significant ST segment depressions were detected throughout the respective study phases.

Maximal exercise performance decreased by 12% during hypoxia compared to normoxia (262.5 ± 45.1 vs. 231.3 ± 81.7 watts), accompanied by a corresponding 10.1% reduction in peak VO2 from 38.6 ± 4.2 to 34.7 ± 7.5 mL/min/kg. In comparison to normoxia, ventricular premature beats (VES) occurred more frequently during exercise in hypoxia (11 VES; mean: 2.8 ± 1.5 VES vs. 60 VES; mean: 15.0 ± 18.7 VES), particularly at lower exercise loads (125.0 ± 134.6 vs. 158.3 ± 82.5 watts; Fig. 2J–K). We observed a higher occurrence of VES during post-exercise recovery in hypoxia compared to normoxia (24 VES; mean: 6.0 ± 6.0 VES; vs. 4 VES; mean: 1.0 ± 1.2 VES).

The datasets that support the findings of this study are available from the corresponding author upon reasonable request.

The key finding of our study is that clinically stable and physically fit post-myocardial infarction patients exhibit QTc interval prolongation in response to hypoxia-induced PH. Additionally, VES occur more frequently during and after exercise, even at altitudes equivalent to approximately 1500 m, likely due to hypoxia-induced PH and sympathetic activation^1^. QTc interval prolongation is also common in chronic PH with impaired right ventricular function^6^. While cardiac output and hematocrit adjustments usually maintain systemic oxygen delivery^1^, microcirculatory dysfunction-induced tissue hypoxia may affect cardiac electrophysiology. There is no evidence of a significant risk of fatal arrhythmias from hypoxia in healthy individuals^1^. However, in patients with heart diseases, QTc interval prolongation and VES should be taken seriously^7^.

Limitations include the small sample size consisting of only four participants and the exclusive inclusion of male participants, which restrict the generalizability of our findings to a broader post-myocardial infarction population. Additionally, asymptomatic electrocardiographic changes may not directly predict clinical outcomes. Moreover, variability in patients' ejection fractions has the potential to introduce heterogeneity in the study population. Furthermore, the inclusion of a patient with NSTEMI, characterized by distinct pathogenesis and myocardial scar mass compared to STEMI, could confound result interpretation. Given the pilot nature of this study, our preliminary findings require careful consideration.

Despite these limitations, our study indicates that even exceptionally healthy, fully revascularized post-myocardial infarction patients may be susceptible to hypoxia-induced QTc interval prolongation and VES, particularly during physical exertion. Albeit the clinical implications of these findings may warrant further investigation, we recommend cardiovascular evaluation by a high-altitude medicine specialist for post-myocardial infarction patients planning high-altitude physical activities. Future research should investigate the efficacy of hypoxia-induced cardiovascular stress testing in guiding such decisions.
